# Topical Spironolactone in the Treatment of Ocular Graft-Versus-Host Disease

**DOI:** 10.7759/cureus.45136

**Published:** 2023-09-12

**Authors:** Calvin W Wong, Annie A Yang, Chia-Yang Liu, Mitchell A Watsky, Xiaowen Lu, Harrison L Le, Richard W Yee

**Affiliations:** 1 Medicine, McGovern Medical School, University of Texas Health Science Center at Houston, Houston, USA; 2 Ophthalmology, Richard W. Yee, MD PLLC, Houston, USA; 3 Medicine, Baylor College of Medicine, Houston, USA; 4 Ophthalmology, University of Cincinnati College of Medicine, Cincinnati, USA; 5 Cellular Biology and Anatomy, Medical College of Georgia, Augusta University, Augusta, USA

**Keywords:** tear film, cornea, ocular keratitis, dry eye disorder, ocular graft-versus-host disease

## Abstract

Introduction: This two-part study aimed to investigate the therapeutic potential of topical spironolactone in ocular graft-versus-host disease (oGVHD). While off-label use of topical spironolactone has been described in dry eye, its efficacy in managing signs and symptoms of oGVHD remains unstudied. Preclinically, we tested the hypothesis that spironolactone induces corneal lipid synthesis in a mouse model. Clinically, we assessed patient response to spironolactone with a retrospective observational design.

Methods: Both immortalized and primary human corneal epithelial cells were stained with oil red O after 9 days of treatment with spironolactone. C57BL/6 mice were dosed thrice daily with one drop in each eye for 18 days. Corneal tissue was stained with oil red O and BODIPY™.

Twenty eyes with oGVHD, as defined by the International Chronic oGVHD Consensus Group, were studied. Corneal fluorescein staining, lid margin vascularity, meibomian gland obstruction, meibum turbidity, zone A posterior lid margin vascularity, and oGVHD diagnostic criteria severity grading were compared in a pre-post study. Follow-up times ranged from 7 to 21 weeks, with a median time of 12 weeks. Statistical analysis was done with STATA 17 by fitting data to a non-parametric model.

Results: *In vitro* results showed an increased number and density of oil red O staining granules in the treatment group versus control in both primary and immortalized human corneal epithelium. *In vivo*, results showed translation to the mouse model with increased corneal epithelial BODIPY™ signal compared to untreated control. oGVHD patients had improved lid margin vascularity (*p *= 0.046), corneal fluorescein staining (*p* = 0.021), and International oGVHD Consensus Group severity scores (*p* = 0.011) after treatment with topical spironolactone. Minimal adverse effects were noted, the most common being mild stinging lasting less than a minute after instillation.

Conclusion: The improved severity scores, lid margin inflammation, and corneal fluorescein staining after weeks of treatment support the rationale that topical spironolactone may benefit oGVHD. The observed lipid production by the corneal epithelium is thought to contribute to this protective effect against ocular surface erosive disease in oGVHD. A mineralocorticoid receptor antagonist, spironolactone may offer therapeutic benefits in oGVHD while avoiding undesirable side effects of topical or systemic glucocorticoids.

## Introduction

Graft-versus-host disease (GVHD) is a condition that can arise from a hematopoietic stem cell transplant. While there have been significant advances in donor matching, prophylactic treatment, and transplant technology, GVHD remains a significant cause of morbidity and decreased quality of life for stem cell transplant patients, affecting various organs and systems.

GVHD can be broadly characterized as either acute or chronic. While acute GVHD (aGVHD) usually develops three to four weeks post-transplant, chronic GVHD (cGVHD) is classically defined as developing after 100 days. cGVHD is closely linked to thymic dysfunction, consequent development of autoreactive T cells, and upregulation of proinflammatory cytokines [1-3]. Ocular GVHD (oGVHD) arises as a more common symptom of cGVHD, with scleroderma-like changes to the eyelids, entropion, and lagophthalmos [4,5]. An impaired lacrimal function may also lead to aqueous tear film abnormalities, and consequent corneal findings of oGVHD include punctate keratitis, diffuse keratitis, ulceration, melts, and, if left untreated, perforation. Posterior findings of oGVHD include lipid deposits, retinal hemorrhage, angiopathy, and optic disc edema [6]. Tear production is often abnormal, with either excessive reflex tearing or insufficient tear production as measured by the unanesthetized Schirmer tear test.

Current treatment of oGVHD is often identical to conventional dry eye therapeutics and immunosuppressants like steroids, tacrolimus, or sirolimus. However, warm compresses, artificial tears, and topical steroids are often insufficient or inadvisable to stop the development of more severe oGVHD activity, which may present as melting or perforation, pseudomembranous conjunctivitis and/or symblepharon. In these cases, steroid eye drops may prove temporarily useful, but their long-term use is limited by the possibility of intraocular pressure elevation, corneal thinning, or infectious keratitis [7]. Tarsorrhaphy may be recommended as an adjunct to other treatments to promote epithelial recovery in severe cases. Treatment of dry eye secondary to GVHD can be complicated and difficult due to the multifactorial nature of the disease. Thus, patients often fail to achieve symptomatic control despite currently available treatment strategies.

Spironolactone is a potassium-sparing, mineralocorticoid receptor antagonist that is classically used in the setting of heart failure for its diuretic effects and anti-fibrotic activity in renal and cardiac tissue. Because of its anti-androgenic activity, classical off-label applications have been used to treat hormonal acne, hirsutism, and female-pattern hair loss. Off-label use of topical spironolactone for dry eye has improved lid margin inflammation and symptom severity [8]. Thus, this study aims to evaluate the potential of spironolactone as a potential therapeutic for oGVHD.

This was presented in part as a paper presentation by Yang at the 2022 American Society of Cataract and Refractive Surgery (ASCRS) Annual Meeting held in Washington, DC, April 22-25, 2022, and by Wong at the 2023 Association for Research in Vision and Ophthalmology (ARVO) Annual Meeting, held in New Orleans, LA, April 23-27, 2023.

## Materials and methods

This study was partly basic laboratory and partly retrospective clinical. In this study's in vitro basic work, a telomerase-immortalized human corneal epithelial cell line (hTCEpi) was grown on standard culture plates until confluent, as previously described [9]. All cells were grown in Dulbecco's modified Eagle's medium (DMEM, Thermo Fisher Scientific, Waltham, MA) supplemented with FBS (3%), 1% Insulin-Transferrin-Selenium (ITS, Thermo Fisher Scientific), and 40 μg/mL gentamicin (Thermo Fisher Scientific). Cells were subpassaged using trypsin (Sigma, Ann Arbor, MI) digestion, seeded in 35 mm dishes (Thermo Fisher Scientific), and cultured in a humidified incubator at 37°C with 5% CO2. The culture medium was replaced with fresh DMEM medium plus 3% serum every two days. Cells were treated in a culture plate with spironolactone 0.003%, spironolactone 0.015%, or control medium for nine days. For lipid Oil Red O staining, cells were washed with PBS, fixed with 4% PFA for 30 min, washed with 60% isopropanol, and allowed to dry at room temperature (RT). Stock Oil Red O (Cat. No. O1391, Sigma-Aldrich) was prepared (5 g oil red O in 1L isopropanol), and the working solution was prepared by diluting 6 ml stock solution with 4 ml of distilled water. This was allowed to stand for 10 minutes and was filtered into a Coplin jar, which was immediately covered. 1ml of Oil Red O working solution was added to each culture well and incubated at RT for 10 min. The Oil Red O solution was removed, and cells were washed 4 times with ddH2O. Images were acquired with an IX73 Olympus microscope (Olympus, New Orleans, LA).

Primary human epithelial cells were obtained from three de-identified donor corneal rims courtesy of Dr. Amy Estes from the Department of Ophthalmology, Medical College of Georgia, Augusta University, and The Eye Guys, Eye Physicians and Surgeons of Augusta, GA. Primary corneal epithelial cell cultures were established using an explant culture method [10]. Briefly, the corneal rim was visualized under a dissecting microscope to confirm that it contained viable epithelial cells. If so, it was washed with Ca++ free PBS (pH 7.2), cut into 1-2 mm fragments, and placed epithelial side up in a 35 mm dish. After approximately 5 minutes in a laminar air-flow culture hood, corneal fragments became attached to the culture dish, and 1.5 mL of DMEM with 10% serum containing 40 μg/mL gentamicin, 1% ITS, and 100 ng/mL cholera toxin (LIST Biological Laboratories, Inc., Campbell, CA) was added and the pieces were cultured in a humidified incubator at 37°C with 5% CO2. The culture medium was replaced every two days. After 7 to 10 days, cells were nearly 100% confluent. Cells were passaged using 0.25% trypsin, centrifuged at 500g for 5 minutes, and subcultured in DMEM with 10% serum containing 40 μg/mL gentamicin (Life Technologies, NY), 1% ITS (Fisher Scientific, Waltham, MA), and 100 ng/mL cholera toxin (LIST Biological Laboratories, Inc., Campbell, CA). Cultured cells were inspected before use under the microscope to ensure minimal contamination by stromal cells.

In vivo testing involved the use of topical spironolactone in C57/BL6 mice dosed with topical spironolactone. A 5 mM stock solution of BODIPY (ThermoFisher Scientific) in DMSO was prepared and stored at −20 °C. 20 μM BODIPY staining solution was prepared by diluting stock solution in PBS. Mice were treated with 1 drop of spironolactone (0.0005%) twice daily for 8 weeks. Mouse eyes were flash-frozen in liquid nitrogen and embedded in an optimal cutting temperature compound (Tissue-Tek, Sakura Finetek, Torrance, CA). Corneas were cryosectioned and incubated for 20 minutes with 20 μM BODIPY and washed 3 times with PBS. The sections were fixed for 10 minutes at room temperature and washed 3 times in PBS. The fixed sections were mounted in Fluoroshield™ with DAPI (Sigma-Aldrich, St. Louis, MO) and covered with glass coverslips. Cryosections were examined using a Zeiss LSM 780 inverted laser-scanning confocal microscope (Zeiss Microscopy, Jena, Germany). All animal studies were undertaken following the guidelines of the ARVO Statement for the Use of Animals in Ophthalmic and Vision Research and were approved by the Augusta University/Medical College of Georgia IACUC committee (#2013-0581).

The clinical aspect of this study is a retrospective case series following the off-label use of spironolactone in 10 patients with chronic oGVHD, defined as "Probable" or "Definite" by the International Chronic oGVHD Consensus Group [11]. The Consensus Group severity score components were recorded at each visit, including the Ocular Surface Disease Index (OSDI), conjunctival injection, Schirmer's unanesthetized tear test, and corneal fluorescein staining. Other clinical signs of ocular surface disease associated with the lid margin included vascularity, zone A vascularity [12], and meibomian gland obstruction and turbidity, routinely recorded on a scale from 0 to 4. These clinical signs from both eyes were included for statistical analysis in a pre-and post-treatment design. Exclusion criteria included previous use of topical spironolactone, systemic steroid treatment changes, and other concurrent confounding ocular surface diseases such as Sjogren's syndrome, infectious keratitis, or severe lagophthalmos. After starting spironolactone, 0.0005% in both eyes four times daily, follow-up time ranged from 7 to 21 weeks, with a median time of 12 weeks. Statistical analysis was performed using the Wilcoxon sign rank test in STATA 17. This study followed the Declaration of Helsinki and under institutional review board exemption criteria.

## Results

Lipid demonstration in cultured cells

**Figure 1:**
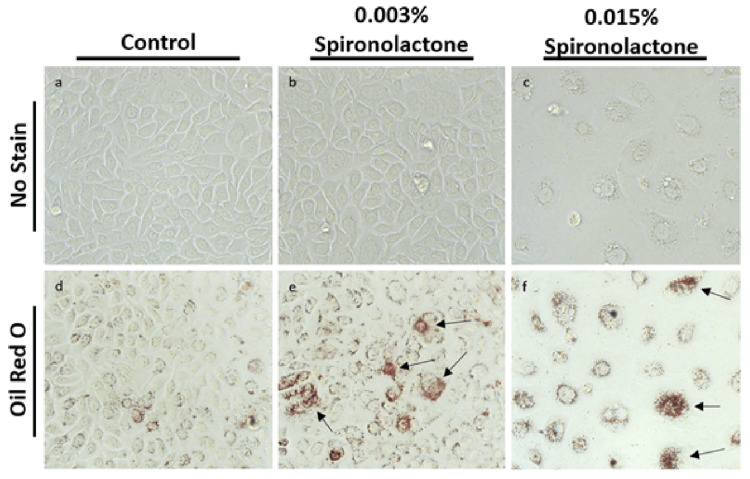
hTCEpi treated with control, 0.003% spironolactone, and 0.015% spironolactone. (a-c) Immortalized human corneal epithelium without oil red O shows no staining. In panel (c), loss of cell confluency and survival is noted in the group treated with 0.015% spironolactone. (d) Cells treated with control demonstrate baseline oil red O staining. (e) Cells treated with 0.003% spironolactone demonstrate diffusely increased staining in addition to focal, intensely staining lipid granules indicated by arrows. (f) At spironolactone dosage of 0.015%, a toxic effect is noted with decreased cell survival, loss of confluence, abnormal size and morphology. Arrows indicate concentrated granular staining.

hTCEpi treated with control, 0.003% spironolactone, and 0.015% spironolactone are shown in Figure 1. Cell morphology was similar to controls at lower dosages, with retained confluence, similar shape and size, and comparable survivability (Fig 1c, 1e). Increased intensity of granular staining was noted in treated tissues (Figs 1e, 1f) compared with control tissue (Fig 1d). At higher concentrations of 0.015% spironolactone, cell survival was decreased, and cells showed morphological changes such as interrupted connections and enlargement (Fig 1c, 1f).

**Figure 2:**
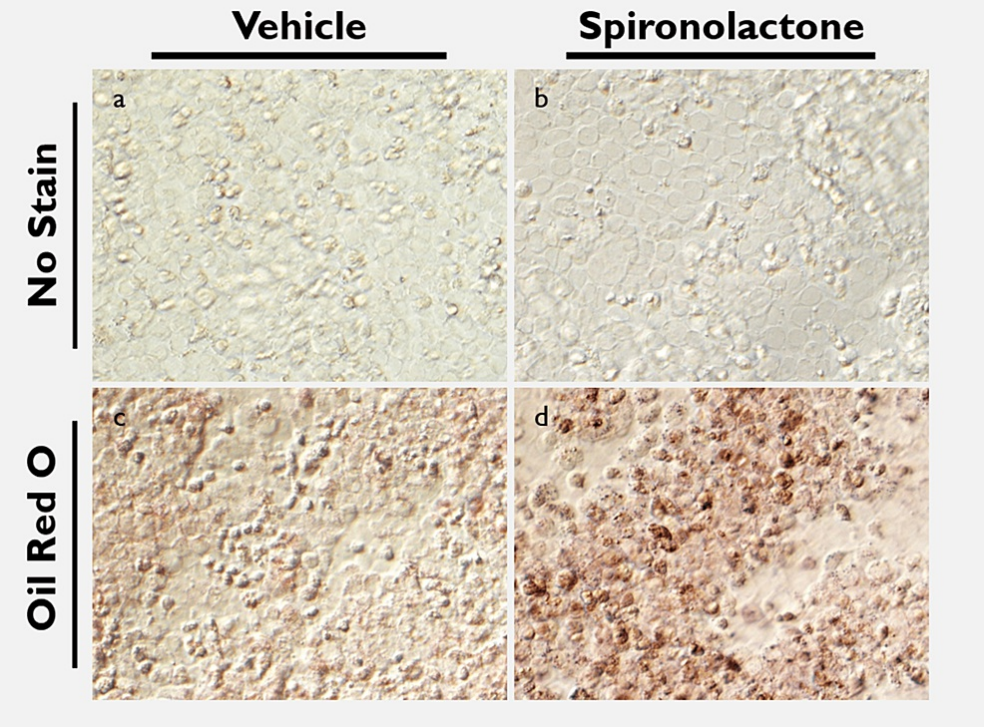
Oil red O staining of primary human corneal epithelium treated with spironolactone versus control. (a, b) Primary human corneal epithelium shows similar size, confluence and morphology in cells treated with control and with 0.0005% spironolactone. (c) Primary HCE stained with oil red O shows background staining level in tissue treated with control. (d) Increased distribution of oil red O stain in treated tissue compared with control is noted in addition to densely staining bodies within cells. Cellular confluence and morphology remains largely similar between both stained control and treated groups.

Primary HCE treated with 0.0005% spironolactone and with control are shown in Figure 2. Cell survivability was comparable between control and spironolactone groups, with similar observed morphology, cell size, and confluency (Figs. 2a, 2b). Increased oil red O staining was noted in the treated cells (Fig 2d) compared to control (Fig 2c). Further, the presence of darkly staining bodies within cells and increased granular staining was noted in treated tissue (Fig 2d).

Lipid demonstration in mouse models

**Figure 3:**
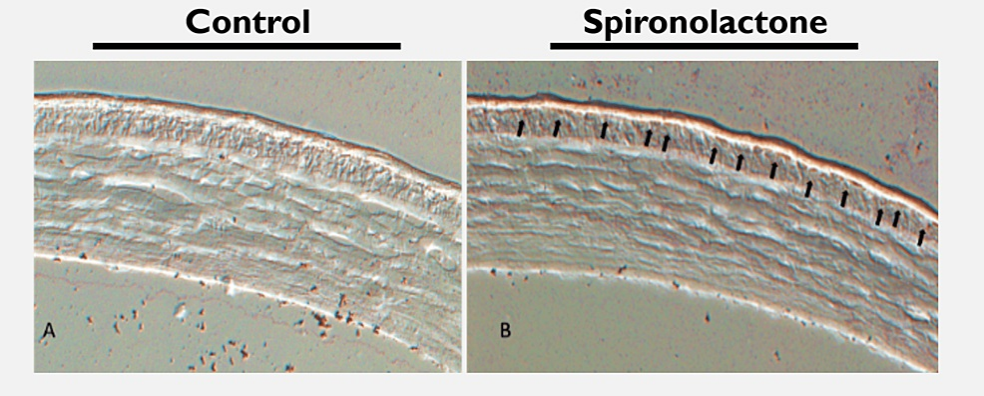
Mouse cornea stained with oil red O and treated with spironolactone versus control. (a) Mouse cornea treated with control, cryosectioned, and stained with oil red O demonstrates baseline stain. Epithelium and stroma are well visualized. (b) Mouse cornea treated with 0.001% spironolactone was cryosectioned and stained with oil red O. Corneal anatomy remains grossly similar to panel (a). Diffuse, slightly increased staining is noted in the epithelium, indicated by arrows.

In vivo, mouse corneas treated with 0.001% spironolactone and control corneas are shown in Figure 3. The corneal structure appeared grossly similar, with increased staining along the epithelium denoted by arrows in Figure 3b.

**Figure 4:**
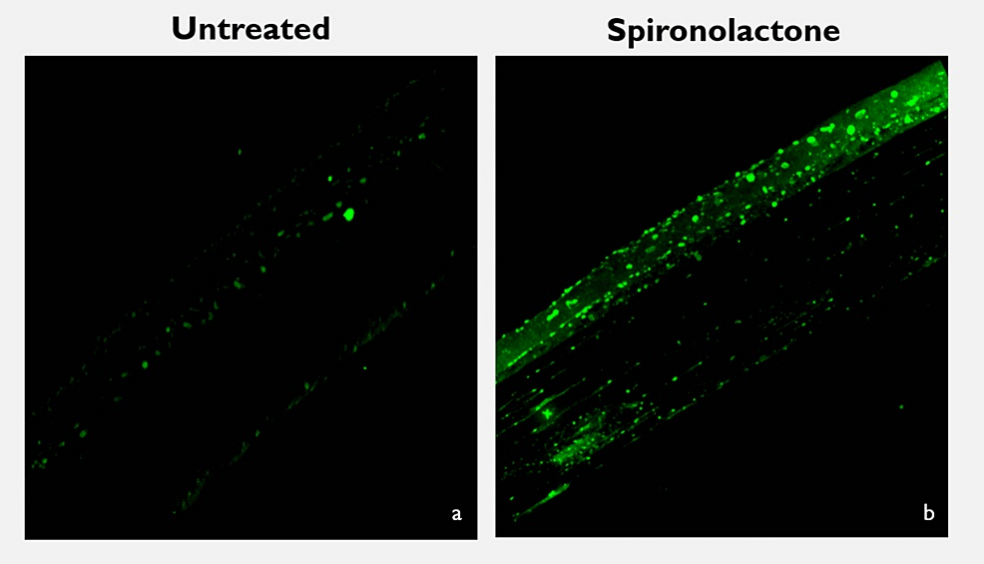
BODIPY staining of mouse corneas treated with spironolactone versus untreated corneas. BODIPY (493/503) staining of untreated mouse cornea shows minimal foci of fluorescent dye. (b) In a similar orientation, mouse cornea treated with 0.0005% spironolactone shows increased distribution of fluorescence throughout the epithelium. Increase in size and number of fluorescent foci are noted relative to untreated tissue in panel (a).

Mouse corneas stained with BODIPY™ fluorescent dye demonstrated lipid granule distribution throughout the epithelium and into the stroma in the spironolactone-treated corneas treated relative to untreated corneas (Figure 4). 

Retrospective study of human subjects

Of the subjects that met the criteria, there were 5 males and 5 females. Age ranged from 38 to 74, with a median age of 64. The cohort was 60% Caucasian, 20% Hispanic, and 20% Asian. After starting topical 0.0005% spironolactone four times a day, statistically significant reductions in corneal fluorescein staining (p = 0.02), lid margin vascularity score (p = 0.045), and Consensus Group severity score (p = 0.011) (Table 1; * representing p < 0.05). Statistically nonsignificant reductions were also noted in the OSDI score and zone A vascularity score. Conjunctival injection, Schirmer's score, and meibomian gland parameters did not show significant change.

**Table 1:** Statistically significant reductions in Consensus Group severity score, corneal fluorescein staining score, and lid margin vascularity score were achieved with spironolactone treatment (all p <0.05).

	Pre-treatment	Post-treatment	p-value
Consensus Group Severity Score	5.5 (SD = .56)	4.1 (SD = .53)	0.011*
Corneal Fluorescein staining	.75 (SD = .12)	.35 (SD = .11)	0.021*
Ocular Surface Disease Index (OSDI)	21 (SD=20)	11 (SD= 10)	0.23
Conjunctival injection			
Right Eye	.33 (SD = .5)	.22 (SD=.44)	0.31
Left Eye	.33 (SD = .5)	.22 (SD =.44)	0.31
Schirmer’s unanesthetized score (mm)			
Right Eye	5.3 (SD=5.5)	6.0 (SD = 7.72)	0.36
Left Eye	6.8 (SD=7.7)	9.1 (SD = 8.2)	0.15
Vascularity score (V)	1.75 (SD = .22)	1.35 (SD = .59)	0.045*
Meibomian Gland Obstruction (O)	2.2 (SD = .29)	2.25 (SD = .32)	0.577
Meibum Turbidity (T)	2.17 (SD = 1.0)	2.0 (SD = .84)	0.331
Zone A vascularity (ZA)	1.55 (SD = .14)	1.45 (SD = .17)	0.157

In all patients, no abnormal increases in intraocular pressure were noted, and the most common side effect reported was a mild stinging sensation lasting no longer than 30 seconds after self-administration of the topical spironolactone.

## Discussion

The increased oil red O staining of cultured human corneal cells and mouse corneas treated with spironolactone and BODIPY™ staining of in vivo spironolactone-treated mouse corneas provides qualitative support for the theory that administration of topical spironolactone is associated with increased lipid production. The association between spironolactone and increased lipid presence in corneal epithelium presents a novel approach to the treatment of dry eye disease and oGVHD, and increased lipid production or presence in corneal epithelium may have a protective effect in cases of oGVHD with prominent evaporative features. Nonclassical off-label effects of spironolactone in the eye have included inhibition of corneal neovascularization, macrophage inhibition, and improved epithelial recovery after insult [13]. Mineralocorticoid receptor blockade has also been associated with upregulation of PPARγ and TGFβ [14], with anti-fibrotic and anti-inflammatory effects noted most prominently in heart and kidney tissue [15]. The clinical implication of PPARγ upregulation with mineralocorticoid receptor blockade remains unclear, though PPARγ has been closely linked with the modulation of lipid metabolism [16].

Further quantitative work is needed to establish a better link between mineralocorticoid receptor blockade-induced PPARγ upregulation and increased lipid production and presence findings. Additionally, mineralocorticoid receptor antagonism has been documented in association with as high as 75 differentially expressed genes [17], so it is unlikely that PPARγ is the only mediator of lipid metabolism in the cornea. The effects of topical spironolactone on the lipidomics of epithelial tissue and the composition of the tear film also remain poorly understood, and future work should explore the quality and localization of lipids in spironolactone-treated corneal tissue.

**Figure 5:**
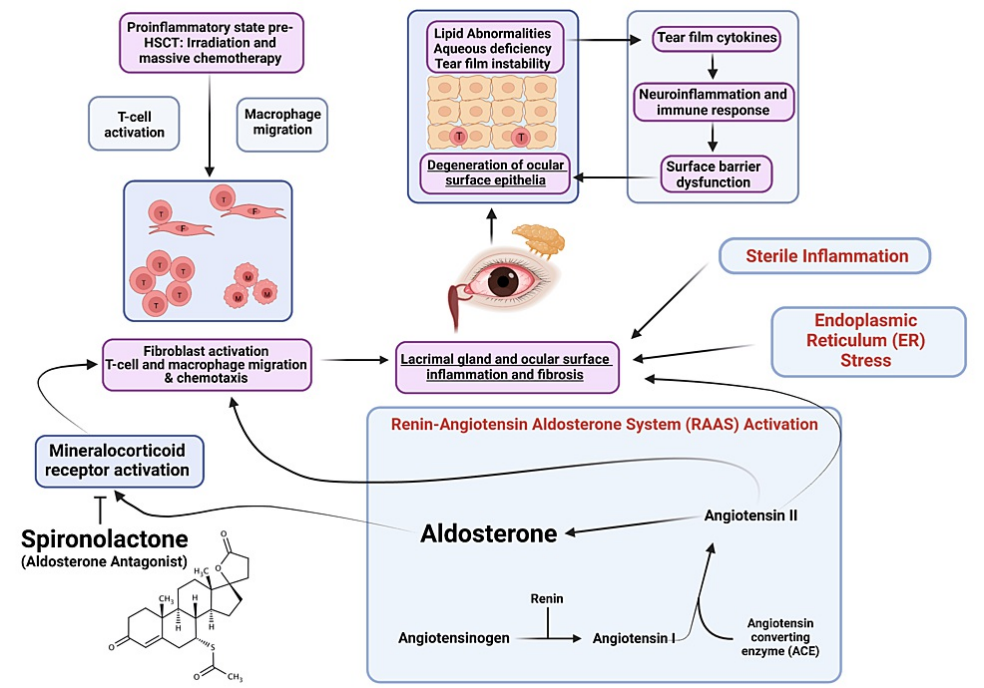
Schematic of ocular GVHD pathophysiology. Fibroblast and lacrimal dysfunction contribute to cyclic lipid abnormalities, keratitis, and inflammation. Spironolactone may have therapeutic benefits by competitive inhibition of the mineralocorticoid receptor and subsequent inflammatory fibrotic processes. Adapted with permission from Ogawa [23].

Blockade of the larger renin-angiotensin-aldosterone system (RAAS) has been shown to have anti-fibrotic effects in ocular tissue [18]. Topical angiotensin receptor blockade has been shown to be associated with inhibition of decreased opacity, corneal scarring, and decreased myofibroblast activity after descemetorrhexis in animal models [19]. Further, the presence of RAAS components has been documented throughout the eye. Expression of angiotensin-converting enzyme isoforms [20], angiotensin II, and angiotensin II receptors have been demonstrated in lacrimal gland tissue in mice [21]. Bulbar conjunctiva and cornea also express angiotensin II and angiotensin-converting enzyme [20], further providing rationale for the observed anti-fibrotic effect seen with upstream RAAS blockade and downstream mineralocorticoid receptor blockade. In a cGVHD mouse model, upregulation of angiotensinogen mRNA in lacrimal glands has been demonstrated [21], further implicating RAAS components in the pathophysiology of ocular GVHD. As pathogenic fibrosis and inflammation are thought to be closely linked to the development of the sicca syndrome in oGVHD [22], RAAS and mineralocorticoid receptor blockade present novel therapeutic approaches in managing this complex disease. The pathophysiology of oGVHD has been described by Ogawa as a cycle of inflammation, fibrosis, and ocular surface pathology arising out of T-cell autoactivity, cellular senescence, tissue renin-angiotensin system (RAS), and endoplasmic reticulum stress (Figure 5) [23]. Treatments targeting the RAS and RAAS, like topical losartan and spironolactone, are directed to disrupt cyclic inflammatory processes that result in surface barrier dysfunction, keratitis, and tear film abnormalities.

The implications of a host of differentially expressed genes in response to mineralocorticoid receptor antagonism remain to be fully understood [17]. However, the growing body of literature documenting the role of the RAAS in the ocular surface and retina suggests that mineralocorticoid receptor antagonism has therapeutic anti-neovascular, anti-fibrotic, anti-inflammatory, and lipogenic qualities in the cornea. Further, mineralocorticoid receptor inhibition has been documented in systemic immunosuppressants such as cyclosporine A and tacrolimus, which are used widely in dry eye disease and GVHD [24]. Topical glucocorticoids remain a mainstay of treatment for acute exacerbations of ocular GVHD. However, long-term use is limited by increased intraocular pressure, infectious keratitis, the development of posterior subcapsular cataracts, and corneal thinning [17,18]. Thus, targeting the RAAS and mineralocorticoid receptors may provide better long-term adjunctive therapeutic options for chronic ocular GVHD.

The statistically significant finding of improved corneal fluorescent staining in this retrospective case series provides clinical context for previous work documenting the association between spironolactone and epithelial wound healing [13,17]. Although there was no comparison group, untreated ocular GVHD-related keratitis is often persistent, with limited improvement or sometimes exacerbation of epithelial insult [25]. The finding of improved lid margin vascularity and hyperemia is also consistent with documented anti-inflammatory, anti-fibrotic qualities of spironolactone. While the Consensus Group severity score was originally designed for use in the context of diagnostic criteria for oGVHD, its utility in tracking inflammation, keratitis, aqueous deficiency, and symptomatology is reflected in the statistically significant reduction in response to spironolactone treatment.

Limitations of this study include the small size of the oGVHD patient sample, its retrospective nature, and the lack of a comparator or placebo group. Further prospective work with larger sample sizes and parametric statistical testing is warranted to better understand topical spironolactone's therapeutic efficacy and safety.

## Conclusions

The findings of this two-part study suggest that topical spironolactone holds therapeutic potential for ocular graft-versus-host disease. The in vitro and in vivo experiments demonstrated increased lipid production in corneal tissue treated with spironolactone, indicating a potential protective effect against ocular surface erosive disease in oGVHD. The retrospective case series of oGVHD patients treated with topical spironolactone showed improved lid margin vascularity, corneal fluorescein staining, and Consensus Group severity scores. These results support using spironolactone as a mineralocorticoid antagonist for managing oGVHD, offering therapeutic benefits while avoiding undesirable side effects associated with topical glucocorticoids. Further research with larger sample sizes and prospective designs is needed to understand better the efficacy and safety of spironolactone in oGVHD treatment.
